# “I am not promiscuous enough!”: Exploring the low uptake of HIV testing by gay men and other men who have sex with men in Metro Manila, Philippines

**DOI:** 10.1371/journal.pone.0200256

**Published:** 2018-07-06

**Authors:** Jan W. de Lind van Wijngaarden, Andrew D. Ching, Edmund Settle, Frits van Griensven, Rolando C. Cruz, Peter A. Newman

**Affiliations:** 1 Faculty of Public Health, Burapha University, Bang Saen, Chonburi, Thailand; 2 HASH Community Centre, Manila, Philippines; 3 United Nations Development Programme, Bangkok, Thailand; 4 Thai Red Cross AIDS Research Center, Bangkok, Thailand; 5 Division of Epidemiology and Biostatistics, University of California, San Francisco, California, United States of America; 6 Department of Health, Quezon City, Philippines; 7 Factor-Inwentash Faculty of Social Work, University of Toronto, Toronto, Ontario, Canada; Cardiff University, UNITED KINGDOM

## Abstract

The Philippines faces a severe HIV epidemic among gay and other men who have sex with men (MSM). HIV testing uptake remains low. A case series of 12 men from Metro Manila were interviewed to explore barriers to uptake of HIV testing services. Most did not see the need to get tested for HIV despite significant risk, based on the misconception they were feeling well or showed no symptoms. Being of a higher socioeconomic class, feeling morally superior to other gay men, distance of the testing facility, fear of what will happen once infected, fear of HIV- and sexual stigma, fear of side effects of antiretroviral drugs and fear of high health care expenses after testing positive for HIV were key reasons why MSM kept postponing their test. Misconceptions about HIV risk, disease, and treatment and care need to be addressed in order to increase uptake of HIV services in this population.

## Introduction

The Philippines is in the middle of a rapidly expanding HIV epidemic among gay men and other men who have sex with men (MSM), people who inject drugs and transgender people [1]. In June 2016, 841 new HIV cases were reported diagnosed in the Philippines, which was the highest since record-taking began; 95% of new cases were men, and 82% of all cases were MSM. Of the new cases, 23.7% were MSM age 15–24 years. Sixty-three people were reported to have died of AIDS in June 2016 alone, bringing the total number of reported deaths for 2016 to 291; again, the vast majority was MSM [2]. HIV prevalence data and data on HIV testing uptake among MSM in 10 selected Metro Manila cities found a range of HIV prevalence of .45–5.50% (average 2.84%), with HIV testing uptake (defined as “HIV tested in the past 12 months and knowing the result”) ranging from 1–41% across cities (average 20%) [3].

A Department of Health evaluation report on HIV behavior change communication programs commented on counseling practices observed in the Philippines [4]. It identified a widespread practice of counselors and medical personnel handing clients an HIV test result in a sealed envelope, without the counselor being aware of the result. Lab technicians mistakenly believed that they would be breaking confidentiality laws if they informed the counselor of the client’s test result. Hence, no post-test counseling takes place at all in these instances, and linkages to antiretroviral therapy (ART) care services were non-existent.

For HIV testing, parental consent is required up to the age of 18. A recent study on MSM in Iloilo City found that 86.5% of the participants had their sexual debut before the age of 18, with the mean age being 15 years [5]. In other words, the great majority of MSM were already sexually active a long time before they turned 18. The current age of consent law for HIV testing prevents MSM younger than 18 from knowing their HIV status and accessing treatment, care and support if they are positive, because they are highly unlikely to seek their parent’s consent for an HIV test, which would be tantamount to revealing that they are at risk for HIV and, likely, gay. There is an effort by civil society organizations to lower the age of consent for testing to 15 years, but it is uncertain if the Senate will agree, and there is no strong leadership in the government supporting this.

The purpose of this case series research project was to explain why significant numbers of Metro Manila-based MSM currently do not access HIV testing and treatment services [6]. The study, supported by the United Nations Development Programme, was designed using the recently introduced concept of the ‘HIV service cascade’, [7, 8], sometimes referred to as the ‘HIV care continuum’, which outlines a deterministic model of HIV services. It starts with prevention, followed by HIV testing; for those at risk who test negative, re-testing is recommended every 3–6 months. For those testing positive, the HIV treatment and care cascade shows the loss-to-follow-up of people newly diagnosed who manage to a) access a confirmatory test, if required, b) access baseline screening tests, c) initiate ART, and d) remain adherent and are retained on ART until their viral load is undetectable.

The idea behind the study was that by exploring and contextualising problems MSM face in accessing HIV testing, treatment and care services, important lessons for improving such services can be learned, aiming to make HIV services more effective, efficient, equitable and more friendly to MSM.

## Methods

The study was conducted in 4 cities in Metro Manila: Caloocan (population 1.6 million), Manila (1.8 million), Pasay (0.4 million) and Quezon (3 million). The cities were selected to achieve a level of diversity of the HIV response in Greater Manila, with some cities expected to have better-functioning HIV services than others. The study protocol was approved by the National Ethics Committee of the Philippine Council for Health Research and Development. A total of 48 men were recruited in the overall study, 12 at each level of the HIV service cascade (three in each city at each level) using purposive sampling based on their level of access to HIV services in the service cascade. In this paper, 12 of these interviews are presented and analysed, i.e. MSM who had never been tested for HIV, either at the time of interview or for a period of several years prior to having a recent HIV test, often after presenting with HIV related symptoms.

Participants were recruited via participating NGOs and Government clinics in the four cities via the professional network of the second author (ADC), who conducted all of the interviews in Tagalog using a semi-structured open interview template. Interviews lasted on average 30–90 minutes. Before each interview, participants were educated about the study and asked to sign an informed consent form indicating they understood the purpose and process of the study, including their rights to confidentiality and withdrawal from the study. Interviews were conducted at a time and location chosen by the participant, at public locations, for example coffee shops or restaurants. After the interview participants were given information about counselling and information providers, both online as well as offline. The interviewer (ADC) made detailed notes using the interview template form during and right after the interviews, making use of the audio recordings where necessary, and developed summary reports in English for each of the participants. Names of participants and salient details that may identify them were changed in these summary transcripts and in this report to protect their confidentiality, and audio recordings were immediately destroyed after transcription, in line with the study protocol. The first author (DLVW) then conducted thematic analysis of the summary transcripts, using manual coding, listing and ranking techniques. Emerging themes were then discussed and confirmed with the interviewer (ADC) and other authors.

## Results

We have chosen to present the data in a case-series format, referring to a type of medical research study that tracks subjects with a known exposure, such as patients who have received a similar treatment, or examines their medical records for exposure and outcome. Looking at individual cases rather than across-the-board themes has the additional benefit of showing the complexity of each of the participants’ lives, where much of the explanatory power of the study is located. The cases of the eight most salient of the 12 participants are presented in this paper; the remaining four participants’ stories corroborated many of the themes that arose in the other narratives. The men were on average 25.8 years old (range: 19 to 42 years). Three worked in mid-to senior office/corporate jobs; three worked in lower-office jobs; three were teachers or trainers; two were students and one (the oldest participant) was an unemployed manager. Half were born in the Metro Manila area and the others had migrated to Manila at a later age. Three of the men presented in this paper had still never tested for HIV despite considerable risk for HIV infection; the others had, by the time of the interview, been diagnosed but were selected because they had postponed HIV testing for 2 or more years after possible exposure.

The first case, Joey, was 33 and lived in Quezon City. Since having tested once, some five years ago, he declined to go for a new HIV test in the years that followed, despite describing himself as a “serial monogamist” who never had a relationship that lasted longer than a number of weeks, and who did not use condoms consistently. He referred to himself as “very high risk” and commented that “I may very well be positive at this time.” Joey knew several people who worked in the HIV field. He commented:

It’s not that I never wanted to take the test, it’s just I never got around to taking the test. [*Chuckles*] […] I guess there’s that part of me that just totally ignores the importance of testing. I mean I hardly see you or anyone of our friends in the HIV advocacy, things get really busy at work and when I’m not at work, I play.

Joey mentioned that he had “12 or 13 friends with HIV apart from those who died over the past years.” This also had not led him to feel the need to get tested: “No. Stubborn, right? I guess I’m apathetic when it comes to death or maybe I am just good in ignoring things I would prefer to ignore. I don’t know”.

Nicolas, age 42, from Pasay City, currently unemployed, was diagnosed with extra-pulmonary tuberculosis (TB). He said his healthcare provider never told him that an HIV test is recommended for all TB patients. Nicholas said he used condoms infrequently and mentioned several instances of sexual risk-taking. Despite this, he said he never wanted to get tested since he said he was “physically okay”; he was a gym fanatic, which he said was a characteristic of the gay lifestyle. Outreach workers approached him for an HIV test several times over the past years, but he said there was no need because “I knew I was healthy.” Later it became clear to Nicolas that he had several friends who were sick with AIDS. He continued to strongly associate having HIV with signs of physical deterioration typical of AIDS:

I had five friends. The first one had boils all over his body. The other one we visited in [Hospital], he was so thin and had suffered much weight loss. That’s how I knew I was okay. I was healthy and I didn’t have weight loss.

Nicolas met outreach workers regularly in a gay bar in Quezon City. Occasionally, there were mobile testing facilities there, but he still did not want to get tested: “[I]t’s a bar, you know, people, when they see you lined up for an HIV test, they will judge you.” He was also worried about confidentiality and seemed to be irritated by the persistence of HIV outreach workers inviting him for an HIV test: “In order to get rid of the outreach workers, I would take condoms and lube [from them], but I never got tested.” He appeared to question the professionalism of the outreach workers he had met: “There’s this back area at the bar where you can cruise and have sex in the dark. And the outreach workers will be in the dark as well [laughs].” Nicolas knew that oral sex, his preferred sexual behavior, was less risky than anal penetrative sex and he used this knowledge to justify his decision not to get tested—despite having engaged in unprotected anal sex occasionally. While he was younger he had gonorrhea twice, and found out about how to treat it; he bought medicines via the Internet. He never saw a doctor for his symptoms. Nicolas never looked for information on HIV via the Internet; again he explained it pointing to his physical health: “I did not have signs, symptoms or weight loss.” Nicolas was not aware that STI and HIV treatment are available for free at the social hygiene clinics in Greater Manila.

The third case, Drake, age 33 from Quezon City, found out he was HIV positive in September 2015. In the 14 years prior, since he became sexually active at age 17 or 18, he had never sought an HIV test because he always used condoms and usually did not engage in anal sex. He believes he was infected when he used the drug Ice (methamphetamine hydrochloride or crystal Ice) for the first (and only) time, during which he had condomless sex. He mentioned that he felt something wrong about his health a few times since then: “[The] first reason is I could feel something that I couldn’t explain, but I knew that there was something wrong. And second, my best friend had herself tested and I accompanied her.” Drake said that he was worried about his confidentiality at the HIV testing center: “[Other clients waiting to be tested] will know if a person is positive or not based on [his] reaction when he comes out of the post counseling [room].” When Drake tested positive, he told his best friend right away, but she did not believe him: “She said I was such a good actor.” Drake thinks his friend does not want to believe that he is positive; perhaps she would not know how to deal with it.

Case number four, Robert, who was 26 and from Manila City, wanted to be a woman and experimented with hormones while in high school, but then decided that life would be easier as a gay man. He became sexually active from when he was raped at the age of six or seven, and would subsequently never use condoms, as he said he preferred the feeling of being anally penetrated without them. Robert looked for information about HIV online but this did not result in him getting an HIV test. He did not want to be tested because he was worried he would be the only one who was positive in his group of friends; for that reason he preferred, for a long time, not to know. In July 2015 he underwent his first-ever HIV test after being convinced to test by his (gay) boss, who was suspicious about a cough that Robert had and which refused to go away. Robert tested positive for HIV. He said he felt sorry that he had not tested earlier, and seemed to blame himself for it: “I was self-centered before but I didn’t know that being positive can reverse your life, either for good or bad. […] And because I was that self-centered, I always went for whatever made me happy.” Once Robert found out, he decided to tell his siblings, but not his parents “because they are already senile.” However his sister then told his mother that he had “AIDS”: “My mother texted me to go to church, pray a lot, go to Cavite where there is a healing priest there. I told her not to worry.” Robert disclosed his status to his friends and colleagues too, including his staff and his boss, who was an important inspiration for Robert to get tested. Robert had a very positive framework for dealing with HIV:

I didn’t take it as punishment, but I took it as a new challenge. There is nothing new in my life aside from office, work, and partying. But when the virus struck me, I saw this as a reason and as a challenge to live longer.

The fifth case study, Justin, aged 28 from Quezon City, found out he was HIV positive in March 2015 with a CD4 count of just six. He had been in a presumably monogamous relationship with his first boyfriend for a period of 11 years. Initially they used condoms, but in the past two years, according to Justin, they had stopped using them. He had had several symptoms of AIDS-related disease in the months before finally taking the test, including TB of the lymph nodes, and had been in contact with medical authorities to test for many different diseases, including TB, cardiovascular disease and cancer:

I felt there definitely was something wrong. […] That’s the time I started to search online for common symptoms. […] My EENT told me to have my lymph tested to know if it was cancerous […]. That night I posted on my Facebook that I might die tonight. People were aware that all my lab tests were negative. Some friends were even texting me but never directly told me to get tested for HIV, but eventually one of them told me to do so. A very close friend of mine […] told me, “Son, I love you but son of a bitch, get HIV tested as I don’t want you to join the [death] statistics.”

Justin seemed totally convinced that it could not be HIV because of his monogamous relationship with his boyfriend, but also because of his boyfriend’s and his own family’s status as doctors, which led him to classify himself and his boyfriend as “low-risk”:

I was already aware of [the HIV service NGO] and my friends in Malate, like X and Y they were always telling me to be careful, but since they know that my partner and I came from a family of doctors, they were sure that we are safe. Also in my ex’s family, being a family of doctors, HIV was a common discussion or topic.

Justin also thought that the healthy looks of his partner meant he could not have HIV:

I am not that equipped with correct info and my partner seemed healthy based on his physical condition, and there is that trust that [my] partner is healthy, aside from the fact that he is a doctor I had no reason to have doubts.

When Justin finally was tested for HIV for the first time, it was so crowded at the testing center that he turned around and left, saying he felt “afraid and intimidated”; he came back later dressed in different clothes and wearing a cap, fearing to be recognized. When he finally tested positive (in March 2015), his boyfriend of 11 years admitted that he had known that he was HIV positive for the past 5 years. Despite this, Justin was willing to stick with him, but his boyfriend insisted on a break-up:

Even when we were in our [HIV] training [2 weeks before the interview, ADC], I was crying and I was saying I felt bad, not because I acquired [HIV] but I felt bad because after I acquired it from [my boyfriend], he left me.

He saw himself as a “victim of love,” but said his HIV infection was partly his own fault too:

But lately I realized, and it’s funny, I think back, now that I’m taking ARV–I have seen that same bottle before, with my ex! But admittedly that was one of my mistakes, I never asked him anything that connected him to being positive, I didn’t even search online what are those bottles and pills I had seen [him take]. All he said was that medical representatives gave him those pills for trials.

Justin received the test result in the company of a family member, and told his entire family about his HIV status the same day. They responded well:

When I finally blurted out the news, there was that moment of silence and they were all just staring at me. My brother told my parents, “Why, dad? Can’t you give your favorite child a hug? Ma, can’t you hug him? He’s your favorite, can’t you stand up and go over his side and give him a hug?” Then they stood up and gave me a hug and uttered, “We shall fight!” The day after, my Mom accompanied me to [the hospital].

Justin also disclosed his testing HIV positive to colleagues at the place he was teaching and was well-supported; colleagues collected 10,000 *pesos* to help with his medical expenses and over the following months his weight went up from 42 to 56 kilograms: “The support I needed came and I never had to look for it.” Asked why this might be the case, he responded:

I guess it's because I have been a good person, that’s why I have been getting all the support, care and love that I need from them. They were all surprised to know, but they all managed to understand my status and looked at the things I am capable of rather than dwelling on the idea that I am sick and I am limited. My family has been very supportive ever since, and I don’t see a reason for them not to accept me [*smiles*]. My friends and officemates normally entrust their secrets and stories with me, I kept them, didn’t judge them, I guess that's why am getting the same respect and acceptance I have given them.

Note how Justin, by positioning himself as “good” and, indirectly, as an innocent victim of HIV, managed to obtain strong support from family, colleagues and friends. He could navigate the tremendous societal stigma of having HIV, and of being gay, by positioning himself as morally upright. Justin ‘read’ his social environment well, and marshaled societal and family support to keep going in the face of the social and institutional stigma and discrimination that characterizes life for many Filipino MSM living with HIV.

Case number six, Vince, aged 26, from Manila, has been involved in male sex work for short periods of time to make ends meet since he was 22. He was employed as an administrative assistant at the time of the interview. He had not tested since 2014, despite having been at considerable risk for HIV. He said he was not “effective” when using condoms with his partners, meaning that he would lose his erection. One of his ex-boyfriends tested positive recently, and a steady sex partner of Vince whom he described as a client also recently tested positive for HIV; with both his ex-boyfriend and this steady client Vince would not use protection. Vince gave the distance to the testing clinic as a reason not to get tested, and also his work schedule, which he described as “toxic.” He mentioned the long waiting times as a factor that might put people off. He seemed resigned to the fact that he might have HIV: “I guess I’ve long waited for and expected a positive test result”. He had not made plans to get tested at the time of the interview, despite the interviewer’s advice to do so.

Case number seven, Zen, aged 21 from Pasay, grew up in Manila and started working as a fast-food crewmember recently. He became sexually active since the age of 18 and would generally not use condoms. He found sex partners via online gay dating apps and the online messaging services Planet Romeo and WeChat. Commenting on what made him decide whether to use condoms or not, he said: “It depends on the looks of the person, if he looks healthy or when I have been exchanging texts with the person for some time, I would trust him and condoms would not play a role anymore.” When Zen had a persistent sore in his mouth, a former boss who was a nurse told him he should have it checked out and also be tested for HIV. After this he found out he was positive in November 2014. He said two factors explained why he had not taken an HIV test earlier, which were similar to Vince and Nicolas above: he had been “busy” and “the fact that I wasn’t feeling anything bad or odd about myself.” This suggests that for Zen it remained difficult to conceptualize or internalize the idea of a communicable disease without symptoms. The Filipino context of pervasive stigma and discrimination against homosexuality and HIV must also have played a role in maintaining Zen’s denial of his potential HIV infection for several years. Zen has not told anybody about his HIV status except the people he has met via his access of HIV services. He believes his brother, to whom he is close, would understand, but he feels embarrassed and he seemed to judge himself in a moral sense: “[I got HIV] because I didn’t think and took for granted my health and the risks that come with the action”.

The final case, Karl, was 26 years old and was a law student from Vigan who moved to Manila when he was 15. He considers himself to be bisexual but was more sexually active with men than with women. He was worried about being possibly infected “because I am promiscuous,” and he never uses condoms. He was diagnosed with Gonorrhea twice, once infecting his then-boyfriend. Karl kept postponing taking the HIV test:

I had a mindset that after taking the bar [*lawyer’s exams*], I will go for an HIV test. I assumed then that by that time, I would be capable to support and provide for my own means in my treatment on the presumption that I will turn out reactive for HIV.

He was influenced in the decision to postpone his HIV test by information he found on the Internet around 2011. This led him to believe that there would be severe side effects from the HIV medicines, if indeed he would need to go on treatment for HIV:

I refused to get tested then regardless of my sexual activities, because I was still studying. […] I based this decision on the things that I have read in several blogs, specifically those of the drug reactions. I was thinking then that it would be taxing on my part, much more so because I am still financially dependent on my mom. I have no source of income to provide for my medication if it turned out reactive. I also considered the possibility of getting through extreme depression [after testing positive].

Note that Karl was worried about the cost of ARV treatment in addition to medication side effects and depression, and was not aware at the time that this treatment was available for free. Karl found information about how long it would take for him to show symptoms of infection, and used this information to justify postponing the HIV test and onset of treatment:

[A]t that time, I was hoping that my immune system was still doing well. I knew then that complications only appear within 7 to 10 years from infection. I did the math; since I became sexually active, complications would only become a problem come 2019. But I was wrong, as certain complications came in as early as 2014.

His family played a role in postponing his HIV test, not only in terms of fear about losing financial support for his studies, but in his fear about the impact of being a “disappointment” to his mother if he tested positive:

As I started law school, I have also developed the hobby of becoming a constant disappointment to my mom with several subjects that I failed. I did not want to add more salt to the injury so I refused from getting tested as the results may contribute to my infamous list of failures.

A final factor that led him not to get tested was Karl’s worry that he would be unable to carry out his planned profession as a lawyer if it became known that he had HIV. However, his plan to postpone his test until finalizing his law studies could not be carried through, as his health started to deteriorate rapidly. He also suffered from Herpes Zoster, hence decided to take the test in 2014 and tested positive.

## Discussion

A number of reasons for not getting tested can be derived from the findings across case studies of diverse MSM in Metro Manila. The most pervasive reason was not seeing the need to get tested, despite often-significant episodes of risk for HIV, and several participants being confronted with friends or acquaintances acquiring HIV or dying of AIDS. Justifications for not getting tested included being in good physical shape, having a healthy lifestyle, not feeling ill and not losing any weight—an oft-mentioned (mis-)perceived necessary sign of HIV infection. Often participants said they had ‘no time’ or ‘never got around’ to getting an HIV test. This suggests on face value a lack of urgency or significance given to HIV testing; however, it may point to their having limited access to HIV testing services, as well as fears of stigma and marginalization in the event of testing HIV-positive.

One important strategy to address delayed or lack of HIV testing among MSM is to make HIV testing available via community organizations and at entertainment venues. A recent meta-analysis found that community-based testing increases HIV testing uptake and increases the average CD4-count at which clients show up for HIV testing and commence treatment, significantly reducing onward transmission. Community-based HIV testing and counseling linked to prevention and care services should therefore be offered in addition to facility-based services [9].

Moralistic ‘us’ versus ‘them’-thinking emerged as an indirect impediment to HIV testing. Justin, for example, thought that his boyfriend, a doctor, and himself, a medical science teacher, were not the type of person that gets HIV. He had never been tested for HIV until he nearly died of his numerous opportunistic infections. Being monogamous was another reason why Justin and some other participants felt no need to get tested—the rationale being that it is okay to have unsafe sex as long as it is in the context of a monogamous relationship. However, most of the participants were ‘serial monogamists’, i.e. they had condomless anal sex in a monogamous relationship but they stayed with the same partner only for a limited period of time before moving on to the next boyfriend, resulting in considerable risk for HIV infection. It was also unclear whether both partners had actually tested negative for HIV before the decision not to use condoms anymore was taken. These are important issues to address in HIV awareness and prevention campaigns.

While monogamy or ‘being faithful’ is a popular strategy for HIV prevention, especially in conservative countries such as the Philippines, monogamy is rarely promoted among gay men in Western settings, contributing to a lack of evidence for the effectiveness of this strategy [10]. Justin’s boyfriend was unable (or unwilling) to tell Justin that he had slept with someone else and not used a condom, and he continued his sexual relationship with Justin without using condoms even after knowing he had been infected with HIV, perhaps in part because he felt unable to bring the issue up and because Justin seemed like he didn’t want to know. This scenario, undergirded by the general theme revealed of preferring to remain ignorant of one’s HIV status in light of the substantial social risks and stigma associated with testing HIV positive, suggests that the promotion of monogamy as a strategy for HIV prevention may be more apropos of settings where open and frank communication between partners about their sexual relationship(s) exists, including the possibility to ‘confess’ to ‘extramarital’ sexual exploits. In many Asian cultures, including Thailand [11], Cambodia [12], Vietnam [13], India [14] and China [15], sexual communication between intimate partners is virtually absent; the situation in the Philippines seems similar. This would also call into question the promotion of ‘negotiated safety’ (where partners agree on an open relationship where they do not use condoms with each other but use condoms consistently with any casual partners they may have) as a prevention strategy for MSM in the Philippines. The effectiveness of negotiated safety has been critically assessed in other settings as well, in the context of highly nuanced, and selectively effective, partner-based (i.e. serosorting) and behavioral (i.e. strategic positioning) strategies for HIV prevention [16, 17]. That many participants in this study invoked looking good or feeling well as signs of being HIV-negative strongly suggests that complex behavioral or partner selection strategies may be highly challenging to enact effectively in the Philippines.

HIV testing was seen as highly embarrassing for some participants, as they thought it would reveal to their social environment that they had a reason to be worried about HIV, i.e. they may have engaged in ‘immoral’ behaviors/‘promiscuity’. One participant mentioned that he thought he was “not promiscuous enough” to get HIV, showing how HIV is indeed still linked to moral ‘depravity’. Improving general knowledge about HIV and its transmission, as well as promoting HIV testing as an act of wisdom, responsibility and courage could help counter the ways in which this morality gets in the way of HIV testing uptake. Countering the numerous sources of such stigmatizing messages and misinformation, while a monumental task, may be an important strategy for promoting HIV testing.

Fear of what happens if one tests positive was also an important reason to postpone. One participant who kept postponing his test was aware that he had been at risk, but was worried that he would end up being the only person with HIV in his circle of close friends. This is emblematic of how ‘silently’ the HIV epidemic operates in the Philippines; most participants did not know another person with HIV personally, at least not before they became infected themselves. It is important to try to break the silence around HIV and make sure there is wider recognition and knowledge of the fact that currently up to 29 MSM become infected with HIV every day in the Philippines. It is also important to advertise the availability and effectiveness of free ARV treatment to counter widespread fatalism.

Several participants were overly fearful of side effects of HIV medications, having read horror-stories about this online; the underlying fear was that such side effects would lead the family to find out about their status. Most participants were not aware that their chosen strategy—waiting for the first opportunistic infections to occur before considering ARV treatment—could have serious health consequences, especially in case of immune reconstitution inflammatory syndrome (IRIS), during which a paradoxical clinical worsening of symptoms occurs immediately after initiating therapy, leading to increased morbidity and sometimes death [18].

Some of the reasons not to get tested were linked to the actual or perceived characteristics of HIV counseling and testing centers. The most important and most frequently mentioned factor was a worry about a lack of confidentiality/privacy of the test result due to the presence of many other people at these centers. Similar reasons for not getting tested for HIV emerged among MSM in India, many of whom reported being more fearful of being found out to be HIV-positive—and subsequent loss of support or cut-offs from family, friends and partners—than of the physical manifestations of HIV itself [19, 20].

Other participants, especially those with low incomes, found the testing centers located too far away, the waiting times too long, or their opening hours conflicting with their work schedule. It is obvious that HIV testing facilities are not equitable at the moment. Lower-threshold and more private HIV testing modalities must be made available, for example via trained outreach workers, self-testing and by extending opening hours of HIV counseling and testing (HCT) centers. Lowering the opportunity costs for HIV testing is a highly important mechanism for making access to HCT more equitable.

An innovation that has emerged in recent years is to have non-medical outreach workers play a role in HIV service delivery. This can occur by accompanying prospective clients to these services or delivering certain services themselves, usually provision of a screening test for HIV and accompanied referral to HIV services. ‘Accompanied referral’ has been shown in Bangkok to dramatically reduce the number of clients who are lost to follow-up after a positive screening test result [21]. Among MSM in China, accompanied referral led to a higher proportion of HIV-positive MSM receiving their confirmatory test results than in conventional testing efforts and a doubling of those linked to the HIV treatment and care system [22]. Having someone ‘hold your hand’ after an HIV diagnosis who helps traverse the necessary next hurdles in accessing treatment and who can provide accurate information and reassurances about side-effects, costs, and other aspects of treatment may be the surest way forward to increase coverage of HIV services among MSM, especially in the context of pervasive stigma and depression.

The diversity of the men in the study, even in this small sample, shows that it is important that HIV prevention, care and support strategies take socio-cultural aspects such as class, level of education, age and concept of self in terms of gender and/or sexuality into consideration [23]. There is insufficient appreciation for the importance of the ways in which ‘homosexuality’ is understood across different generations, classes, age groups and other societal distinctions across different cultures. Incorporating such differences is likely to result in more effective prevention, care and support interventions.

In addition to the strengths of this study in profiling in depth case studies that illustrate the many barriers to HIV testing from the perspectives and life contexts of MSM in Metro Manila, several limitations should be considered. The men described in this study were recruited using purposive sampling, and hence cannot be considered representative of MSM in Metro Manila. Since most of the men were recruited from existing social support groups for people living with HIV, there may be underreporting in the study of experiences of stigma, discrimination, depression and social isolation characteristic of the broader population of MSM living with or at high risk for HIV infection. It is also likely that men of middle- and higher-class backgrounds were overrepresented as they are likely to be more confident to express their sexual identity in the context of a social support group and in a research study. In addition, during translation from Tagalog to English some data may have been lost; also, since summary reports were used rather than verbatim transcripts, some information may have been missed.

## Conclusions

In conclusion, in order to address this emerging public health crisis, the Philippines should make value-free knowledge about HIV, prevention, treatment, care and support available, and work to confront the multiple stigmas against people living with HIV and MSM. HIV testing should be normalized and made more equitable by promoting community- and entertainment-venue-based HIV testing, testing outside the context of an LGBT community for those who do not identify as ‘gay’, and piloting of self-testing or HIV testing delivered at home or at the work-place by trained volunteers. The opening hours of government-run testing services should be expanded to evenings and weekends. In order to reduce the likelihood that clients will not access treatment after a positive HIV diagnosis and are lost to follow-up, a case management approach should be implemented or expanded, ensuring accompanied referral and follow-up by trained, supportive and non-stigmatizing health care workers or volunteers, especially during the first 3 to 6 months of treatment.

## Supporting information

S1 Checklist (DOCX)
