# Supplementary material for: “I am not promiscuous enough!”: Exploring the low uptake of HIV testing by gay men and other men who have sex with men in Metro Manila, Philippines
Source: PLoS One. 2018 Jul 6;13(7):e0200256. doi: 10.1371/journal.pone.0200256 (PMC6034862; doi:10.1371/journal.pone.0200256)
Supplement: S1 Checklist — (DOCX) [file pone.0200256.s001.docx]

**Consolidated criteria for reporting qualitative studies (COREQ): 32-item checklist**

Developed from:

Tong A, Sainsbury P, Craig J. Consolidated criteria for reporting qualitative research (COREQ): a 32-item checklist for interviews and focus groups. *International Journal for Quality in Health Care*. 2007. Volume 19, Number 6: pp. 349 – 357

**YOU MUST PROVIDE A RESPONSE FOR ALL ITEMS. ENTER N/A IF NOT APPLICABLE**

| **No. Item** | **Guide questions/description** | **Reported on Page #** |
| --- | --- | --- |
| **Domain 1: Research team and reﬂexivity** |  |  |
| *Personal Characteristics* |  |  |
| 1. Inter viewer/facilitator | Andrew Desi Ching (ADC) | Methods, #5 |
| 2. Credentials | Jan W. de Lind van Wijngaarden is the lead researcher and first author and has an MA, MPH and PhD; the interviewer ADC has a bachelor’s degree in marketing and a diploma in counseling, also has a decade of experience as an HIV counselor | N/A |
| 3. Occupation | Lead researcher is foreign expert at Burapha University in Thailand as well as independent consultant; second author is director of an HIV support CBO and works as HIV counselor | Not mentioned. |
| 4. Gender | Both the lead author and the interviewer are gay men | Not mentioned in the document. |
| 5. Experience and training | Lead researcher trained the interviewer for several days before the onset of data collection, this happened face to face in Manila; multiple discussions with key informants were held in the process of developing the research protocol | Methods, #5 |
| *Relationship with participants* |  |  |
| 6. Relationship established | Most research participants already knew the interviewer from his profession as a counselor | Methods, #5 |
| 7. Participant knowledge of the interviewer | This was explained during the informed consent procedure. | Methods, #5 |
| 8. Interviewer characteristics | It is mentioned that the interviewer is a counselor who works in HIV service provision and knew many participants professionally. | Methods, #5 |
| **Domain 2: study design** |  |  |
| *Theoretical framework* |  |  |
| 9. Methodological orientation and Theory | No methodological orientation or theory was discussed. The study can be seen as an exploration to develop theories to explain lack of HIV testing. | N/A |
| *Participant selection* |  |  |
| 10. Sampling | Purposive sampling via the NGO and personal network of the 2^nd^ author (ADC) | Methods, #5 |
| 11. Method of approach | Mainly via online chat-programs and via NGOs and HIV services, as discussed | Methods, #5 |
| 12. Sample size | 48 men were in the overall study looking at 4 levels of the HIV service cascade. Of these 48, the focus of this paper was on a sub-group of 12 who had had difficulty / refused HIV testing. They were in this part of the study; the 8 most salient of them are discussed in the paper | Methods, #5 |
| 13. Non-participation | There were no reports of refusals or non-participation, possibly due to the pre-existing trusting professional relationship between the interviewer and many of the respondents. | N/A |
| *Setting* |  |  |
| 14. Setting of data collection | In public spaces, mainly coffee-shops, chosen by the participant | Methods, #5 |
| 15. Presence of non-participants | Nobody else was present during the interviews | Not mentioned |
| 16. Description of sample | All are gay men living with HIV infection or possibly living with HIV but undiagnosed. | Results, #6 |
| *Data collection* |  |  |
| 17. Interview guide | Questions, prompts, guides were developed by the lead author and used/tested and adapted by the interviewer during the beginning of the process of data collection. | Not mentioned |
| 18. Repeat interviews | No repeat interviews were carried out. | Not mentioned |
| 19. Audio/visual recording | The interview was audio-recorded to help the interviewer in developing his summary report; audio-recordings were immediately destroyed after summary reports were finalised. | Methods, #5 |
| 20. Field notes | Field notes were made during the interview. | Methods, #5 |
| 21. Duration | Interviews lasted on average 30-90 minutes | Methods, #5 |
| 22. Data saturation | Data saturation was not discussed in this exploratory study, but since 8 salient cases were described and discussed in the paper out of a total of 12 interviews conducted in this category, this suggests that data saturation did occur, and that the most important factors affecting late HIV testing were derived from the interviews. | Not mentioned in the document |
| 23. Transcripts returned | Transcripts were not developed; summary reports were made | N/A |
| **Domain 3: analysis and ﬁndings** |  |  |
| *Data analysis* |  |  |
| 24. Number of data coders | The first author coded the data | Methods |
| 25. Description of the coding tree | A description of the coding tree was not made; codes emerged inductively from the data. | N/A |
| 26. Derivation of themes | Themes were inductively derived from the data; they were also informed by the 2^nd^ author, with extensive experience as a counselor, who implemented and iteratively shaped the topic guide/questionnaire based on his knowledge, insights and the emerging interview data. | Methods, #6 |
| 27. Software | No software was used for data analysis. | N/A |
| 28. Participant checking | No feedback from participants was obtained, but two feedback workshops with HIV case managers and other HIV professionals were held to discuss and ultimately confirm the data | Not mentioned in the document. |
| *Reporting* |  |  |
| 29. Quotations presented | Yes, quotations were included and participants were identified with a pseudonym to protect their anonymity. | Results, #6-14. |
| 30. Data and ﬁndings consistent | Si | Discussion, #`4-19. |
| 31. Clarity of major themes | Yes, major themes clearly emerge from the findings and are summarized in the Discussion section | Discussion, #14-19. |
| 32. Clarity of minor themes | Yes | Discussion, #14-19. |

**Once you have completed this checklist, please save a copy and upload it as part of your submission. When requested to do so as part of the upload process, please select the file type: *Checklist*. You will NOT be able to proceed with submission unless the checklist has been uploaded. Please DO NOT** **include this checklist as part of the main manuscript document. It must be uploaded as a separate file.**
